# CRISPR medicine for blood disorders: Progress and challenges in delivery

**DOI:** 10.3389/fgeed.2022.1037290

**Published:** 2023-01-06

**Authors:** Tahereh Mohammadian Gol, Guillermo Ureña-Bailén, Yujuan Hou, Ralph Sinn, Justin S. Antony, Rupert Handgretinger, Markus Mezger

**Affiliations:** ^1^ Department of Hematology and Oncology, University Children’s Hospital, University of Tübingen, Tübingen, Germany; ^2^ Abu Dhabi Stem Cells Center, Abu Dhabi, United Arab Emirates

**Keywords:** CRISPR/Cas, blood disorder, physical delivery, viral vectors, non-viral vectors

## Abstract

Blood disorders are a group of diseases including hematological neoplasms, clotting disorders and orphan immune deficiency diseases that affects human health. Current improvements in genome editing based therapeutics demonstrated preclinical and clinical proof to treat different blood disorders. Genome editing components such as Cas nucleases, guide RNAs and base editors are supplied in the form of either a plasmid, an mRNA, or a ribonucleoprotein complex. The most common delivery vehicles for such components include viral vectors (e.g., AAVs and RV), non-viral vectors (e.g., LNPs and polymers) and physical delivery methods (e.g., electroporation and microinjection). Each of the delivery vehicles specified above has its own advantages and disadvantages and the development of a safe transferring method for *ex vivo* and *in vivo* application of genome editing components is still a big challenge. Moreover, the delivery of genome editing payload to the target blood cells possess key challenges to provide a possible cure for patients with inherited monogenic blood diseases and hematological neoplastic tumors. Here, we critically review and summarize the progress and challenges related to the delivery of genome editing elements to relevant blood cells in an *ex vivo* or *in vivo* setting. In addition, we have attempted to provide a future clinical perspective of genome editing to treat blood disorders with possible clinical grade improvements in delivery methods.

## Introduction

Genome editing technologies have been extensively used in scientific research with the aim of genome modification. Zinc-finger nucleases (ZFNs), transcription activator-like effector nucleases (TALENs), and meganucleases (MegNs) are the previously developed approaches for targeted genetic editing (Gaj et al., 2013; Alagoz and Kherad, 2020; Khalil, 2020). The main disadvantages of ZFNs are low targeting efficacy and reduced specificity, while MegNs have low design flexibility. TALENs have shown to be highly efficient and specific. It is rather their design, assembly and construction that are cumbersome and that have limited their use, than their performance (Xu et al., 2020; Siva et al., 2021). Juillerat et al. modified the TALE scaffold by implementing non-conventional repeat variable diresidue (ncRVDs) and could improve TALEN-mediated specificity to target HBB locus and reduce off-site targeting (Juillerat et al., 2015). The subsequent development of clustered regularly interspaced short palindromic repeats-CRISPR-associated protein 9 (CRISPR-Cas9) as a powerful genome editing tool initiated notable improvement in the field of gene therapy due to its versatility and ease of use.

CRISPR-Cas system is originated from the microbial immune system and its application is more convenient and flexible than other engineered nucleases (Adli, 2018; Siva et al., 2021). Target-specific single guide RNA (sgRNA) and Cas endonuclease are the two main components of the CRISPR-Cas system. sgRNA is composed of custom-designed short CRISPR RNAs (crRNAs) and the scaffold so called trans-activating crRNA sequence (tracrRNA) (Yip, 2020). According to a recently published classification, CRISPR-Cas system has 2 classes, 6 types and 33 subtypes (Makarova et al., 2020), among them type II CRISPR-Cas system is the most frequently used type which uses *Streptococcus* pyogenes Cas9 endonuclease (Rodríguez-Rodríguez et al., 2019). During the editing process, sgRNA makes a complex with Cas9 directing it to the target site. After recognition of the protospacer-adjacent motif (PAM), Cas9, creates a double strand break (DSB) in the target site (Rodríguez-Rodríguez et al., 2019; Zhao et al., 2021). The induced DSBs can be repaired through two main DNA repair pathways in the cells: non-homologous end-joining (NHEJ) which is preferred for making gene knockout and homology-directed repair (HDR) pathway which is important for knock in strategy (Klaver-Flores et al., 2020). Base and prime editing are the recently developed CRISPR-Cas based genome editing mechanism in which DNA can be edited in the target site without the generation of DSBs, avoiding in this way potential genomic rearrangements (Kantor et al., 2020; Antoniou et al., 2021).

The CRISPR-Cas system has various important applications in medicine including identifying genes involved in different diseases, developing disease models, establishing diagnostic and therapeutic approaches, cancer immunotherapy and drug screening (Antony et al., 2018b; Ureña-Bailén et al., 2019; Sun et al., 2020; Asmamaw and Zawdie, 2021). One of the proven applications of CRISPR-Cas in human medicine is its therapeutic potential in blood disorders (Zhang and McCarty, 2016; Daniel-Moreno et al., 2019). Blood disorders include various diseases with abnormalities in different stages of hematopoiesis such as Fanconi anemia, amegakaryocytic thrombocytopenia, β-hemoglobinopathies, hemophilia and cancer (Daniel-Moreno et al., 2019). A critical step in CRISPR-Cas based gene therapy for blood cells is choosing the appropriate delivery strategy to transfer the CRISPR components into the cells. Safety and specificity are two major concerns of applying CRISPR-Cas therapeutics in target cells, particularly in translational medicine. It is crucial that the selected delivery system can efficiently transfer the editing tools to the target cells and lead to high editing efficiency and low off target effects (Lino et al., 2018). This issue is more critical in hematopoietic stem and progenitor cells (HSPCs) because an inaccurate delivery system can introduce genotoxicity and impact their stem cell properties. (Cannon et al., 2021). In this review, we discuss the progress of CRISPR-Cas based gene therapy in blood disorders focusing on the pros and cons of different methods for the delivery of CRISPR components into the blood cells.

## CRISPR-Cas9 components

### Cas9 and sgRNA

**FIGURE 1:**
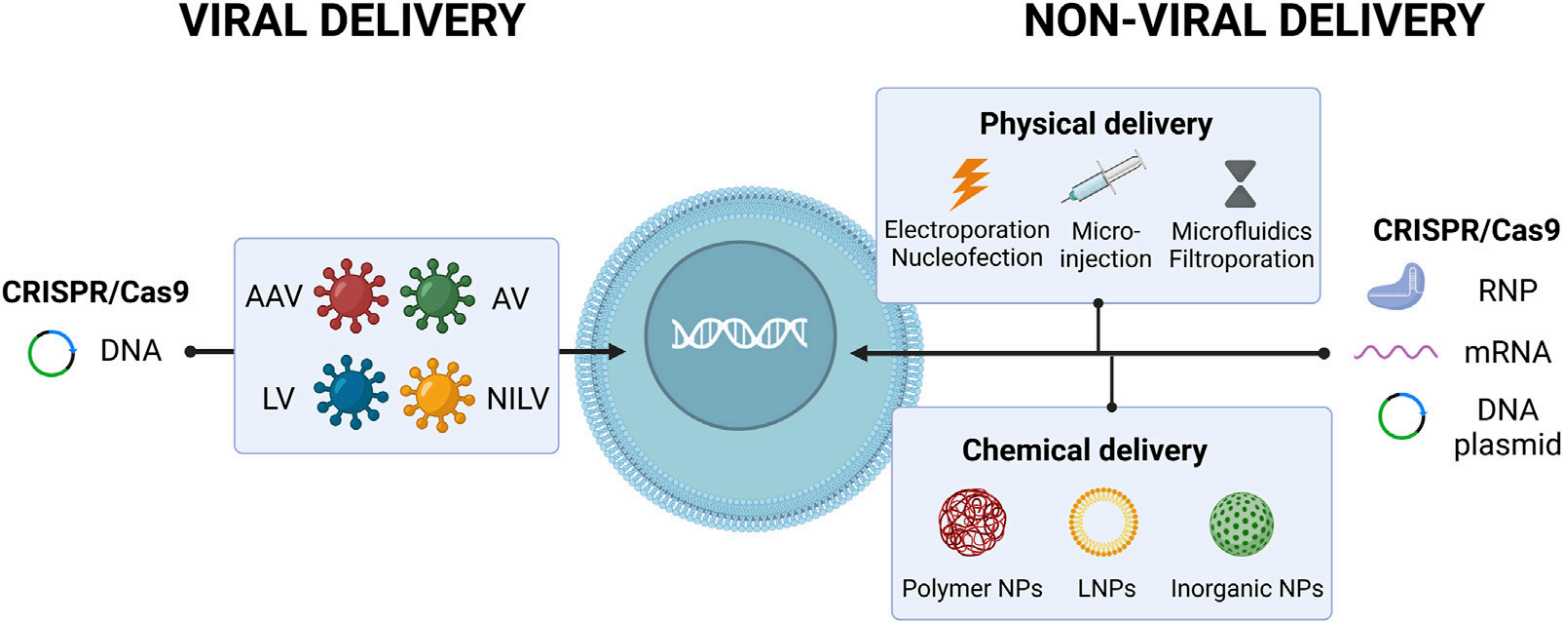
Schematic diagrams of CRISPR-Cas delivery formats and methods. CRISPR-Cas system can be delivered into the cells in DNA, RNA or RNP format. Delivery strategies of CRISPR-Cas9 system are categorized into three groups: physical delivery, chemical delivery and viral vectors.

CRISPR-Cas9 system can be delivered into the cells in three common forms: DNA, RNA and protein (Figure 1). In the DNA format method, one or two plasmids have to be introduced in the nucleus of cells to encode for Cas9 protein and sgRNA (Shalaby et al., 2020). This strategy can increase cellular toxicity in HSPCs due to persistent plasmid expression and induction of undesired mutations (Seema Rani Padhiary, 2021). Lattanzi et al. (2019) showed that using CRISPR-Cas9 plasmids to target hereditary persistence of fetal hemoglobin (HPFH) like region in HSPCs leads to a high frequency of genomic rearrangements (about 30%) and reactivation of γ-globin gene expression but also induce significant cell toxicity. In the RNA form, Cas9-encoding mRNA and the sgRNA can be delivered to the cell at the same time. In this method, low stability of RNA may result in low editing efficiency (Hendel et al., 2015). On the other hand, there is no risk of genomic insertional mutagenesis and the transient expression favors less off-target activity. Since Cas9 mRNA does not need to enter the nucleus, the editing process is also accelerated (Antony et al., 2018a; Behr et al., 2021). Transferring Cas9 protein and gRNA as an RNP complex can solve molecular stability issues, providing high editing efficiency and low toxicity due to immediate gene editing (González-Romero et al., 2019; Yip, 2020).

### Donor template

When the aim of gene editing is to correct a mutation or insertion of a new sequence to the genome, the CRISPR system requires an additional component, the so-called donor DNA template or repair template. Donor templates can be delivered to the cells in plasmid form, synthetic double-stranded DNA oligonucleotide (dsODN), synthetic single-stranded DNA oligonucleotide (ssODN) or viral vectors (Behr et al., 2021). Design and production of dsODNs, ssODNs is faster, simpler and more cost effective than other formats (Romero et al., 2019). Although it is recommended to use the ssODN for the insertion of short sequences and plasmids for larger ones (Song and Stieger, 2017) the perfect donor template format is not clearly known yet. Various factors are involved in ODN design and can influence the efficiency of HDR including size, orientation, length of homology arms and symmetry. ssODNs are usually designed by ≥30 nucleotides homology arm at both sides of the Cas target site (Zhang et al., 2022). Efficiencies between performing HDR with shorter ODN to introduce smaller mutations or long HDR templates have challenges, especially in terms of viabilities because longer ssODNs can cause cell cytotoxicity (Okamoto et al., 2019). Moreover, it has been shown that using longer dsDNA to introduce larger sequences is less efficient due to their size which makes their transfer to the cells more difficult. Long dsODN also can negatively affect cell viability by induction of concatemers in eukaryotic cells (Ghanta et al., 2021). Romero et al. compared Adeno-associated virus type 6 (AAV6) and ssODN application as donor template for CRISPR-Cas9 mediated editing of the mutation responsible for sickle cell disease (SCD) in *HBB* gene. The result demonstrates that using AAV6 as donor template gives rise to higher HDR rates (between 50% and 60%) *in vitro* in comparison to ssODN. However, *in vivo* analysis in immunodeficient mouse xenografts showed similar frequency for AAV6 and ssODNs. Moreover, despite of low toxicity *in vitro*, AAV template negatively affect the *in vivo* engraftment of the HSPCs (Romero et al., 2019). In another study, Ferrari et al. (2022) compared template delivery between integrase defective lentiviral vector (IDLV) and AAV (ssAAV2/6 and other AAV genome forms), showing that delivery *via* IDLV in HSPCs mitigated genotoxic burden, thus confirming the intrinsic issues when using AAV. Nguyen et al. added truncated Cas9 target sequence (tCTSs) to the end of HDR template to facilitate transferring of the template to the nucleus through interaction with RNP and could increase knock-in efficiency (Nguyen et al., 2020). To improve this method by decreasing the cell toxicity of dsDNA, Shy et al. developed a hybrid HDR template by cooperating of a long ssDNA and short dsDNA including CTS on both sides. Applying this method in combination with HDR enhancing molecules in different genetic loci and various types of primary hematopoietic cells resulted in enhanced knock in efficiency and yield (Shy et al., 2022).

## Delivery methods

The ability to deliver gene editing components safely and efficiently into the cells is a critical issue for successful gene therapy. In general, gene editing components such as nucleases and guide RNAs can be delivered into the cells through three strategies: physical delivery, chemical delivery and viral vectors (Figure 1). Each method has highlights and challenges. Choosing the appropriate delivery system depends on the status of the experiment (*in vitro* or *in vivo*), type of cargo (DNA, mRNA, or protein) and the targeted cell or organ type. Moreover, different technical challenges including efficiency, specificity, risk of insertional mutagenesis and immune response induction have to be considered in this context (Yang et al., 2021).

### Physical delivery

Electroporation is an electro-physical, non-viral and fast method for the delivery of exogenous materials into the cells and tissues. In this method, an electric field is utilized to disturb the phospholipid bilayer of the membrane, thereby inducing the formation of temporary pores which allow the delivery of external molecules into cells (Bao et al., 2010; Yip, 2020). Electroporation is safer and more economical compared to viral methods, however, when it is not properly optimized, it can lead to cell death, especially in stress-sensitive cells. The major advantage of electroporation is its applicability for different types of cells including blood cells (Yip, 2020) although in case of using high intensity pulses may lead to changes in the properties of HSPCs (DiTommaso et al., 2018). Electroporation is widely in use for the delivery of CRISPR components into the blood cells *ex vivo*. In this strategy, after electroporation of the CRISPR components, *ex vivo* edited hematopoietic stem/progenitor cells of the patients are transplanted back. Vuelta et al. (2020) used electroporation for the delivery of the Cas9 RNP complex for disrupting BCR/ABL1 oncogene in leukemic stem cells. Transplantation of *ex vivo* edited cells restored normal hematopoiesis in NSG mice. Other preclinical studies also reported promising results after using electroporation for *in vitro* delivery of CRISPR for the treatment of blood disorders (Xie et al., 2014; Smith et al., 2015; DeWitt et al., 2016). Electroporation is an acceptable method in CRISPR-based cancer immunotherapy for *in vitro* and *ex vivo* manipulation of immune cells including T cells, B cells and natural killer (NK) cells (Naeimi Kararoudi et al., 2018; Wu et al., 2018; Afolabi et al., 2019; Greiner et al., 2019). More importantly, several clinical studies are ongoing using electroporation for CRISPR based gene editing in blood disorders (Table 1). CTX001, a CRISPR gene-edited therapy, for the treatment of β-thalassemia and sickle cell disease, is in phase 2/3 of clinical trial (NCT03655678 and NCT03745287). In these clinical trials, hematopoietic stem cells are electroporated with CRISPR-Cas9 to target the BCL11A gene and demonstrated to produce high levels of fetal hemoglobin (Frangoul et al., 2021). Nucleofection, a modified form of electroporation for direct delivery of nucleic acids into the nucleus of different cells, has also been proven to be an effective way of transfecting human CD34^+^ cells (von Levetzow et al., 2006; Antony et al., 2018b; Vaidyanathan et al., 2018).

**TABLE 1:** Clinical CRISPR based gene therapy trials using electroporation as delivery system.

Disease	Target cell	Target genes	Intervention	Phase	NCT
Beta thalassemia	HSPC	*BCL11A*	CTX001	1/2	NCT03655678
Sickle cell disease	HSPC	*BCL11A*	CTX001	2/3	NCT03745287
Sickle cell disease	HSPC	*HBG1/HBG2*	EDIT-301	1/2	NCT04853576
Leukemia/Lymphoma	T cell	*HPK1*	XYF19 CAR-T cells	1	NCT04037566
Leukemia/Lymphoma	T cell	*TCR*, *B2M*	UCART019	1/2	NCT03166878
Multiple myeloma	T cell	*TCRα, TCRβ, PD-1*	NYCE T cells	1	NCT03399448
B cell lymphoblasts leukemia	T cell	*TRAC*, *CD19*, CD22	CTA101	1	NCT04154709
B-ALL	T cell	*CD52*, *TCRαβ*	PBLTT52CAR19	1	NCT04557436
T-ALL	T cell	*TRAC*	WU-CART-007	1/2	NCT04984356

Microinjection is another physical delivery method in which genome editing components can be directly injected into cells under a microscope using very small needles (Elaswad et al., 2018). This method is suitable for *in vitro* and *ex vivo* delivery of the CRISPR system and is used mainly for embryonic genome editing and the creation of transgenic animal models (Horii et al., 2014; Ma et al., 2014). Microinjection can be considered a potential method for CRISPR delivery in human blood stem/progenitor cells as delivery of macromolecules into the HSPCs without negative effect on cellular function is previously shown (Anderson et al., 1980; Davis et al., 2000). However, the processing of only one cell at a time can make the procedure more labor and time-consuming in comparison to other delivery methods.

The microfluidic device is a membrane deformation-based delivery system that uses physical constriction to change the shape of the cell and create transient pores in the cell membrane. Consequently, the crossing of a variety of biomolecules such as CRISPR components by passive diffusion is enabled (Zhang et al., 2021). Ma et al. (2017) developed a specific microfluidic chip for HSPCs. This Nano-Blade Chip (NB-Chip) is designed using silicon instead of polydimethylsiloxane (PDMS). Interestingly, using NB-Chip for transferring macro-molecules or plasmids into the HSPCs was more effective than electroporation in terms of longer persistence of HSPCs’ inherent pluripotency. They could deliver CRISPR in RNP complex format to the human HSPCs and disrupt CCAAT/enhancer-binding protein-α (C/EBPα/CEBPA) p42 *in vitro*.

Filtroporation is another approach for the delivery of CRISPR system in HSPCs. In this method, cells are forced to pass through microporous membranes to increase the permeability of the cells (Stewart et al., 2018; Zhang et al., 2021). Transmembrane internalization assisted by membrane filtration (TRIAMF) is based on the filter membrane cell permeabilization technique to deliver the RNPs to CD34^+^ HSPCs (Yen et al., 2018). Using this system, Yen et al. (2018) could induce indels in the γ-globin gene in HSPCs *in vitro* (44% for HBG2 and 33% for HBG1 site). *Ex vivo* TRIAMF/RNP treatment of HSPCs did not change the engraftment competency and multilineage potential in (SCID)/Il2rg^−^/^−^ (NSG) mice.

### Chemical delivery

Chemical vectors are the other alternative for the non-viral delivery of CRISPR components into the cells. These methods are safer than viral vectors and do not apply much stress on cells in comparison to physical delivery (Yip, 2020).

Lipid materials such as liposomes and lipid nanoparticles (LNPs) provide a safe and efficient delivery method for nucleic acids. Due to their negative charge, nucleic acids are not able to enter the cells through the membrane but their encapsulation into liposomes eases the way for crossing the membrane (Pensado et al., 2014). In a recent study, lipid nanoparticles are used for the delivery of Cas9 mRNA and guide RNA to target antithrombin in hemophilia mouse models (Han et al., 2022). Antithrombin is a thrombin inhibitor and anticoagulant encoded by the serpin family C member 1 (*SERPINC1*) gene. Reduction of antithrombin level is important for balancing coagulation and hemostasis in hemophilia (Sehgal et al., 2015). Han et al. (2022) could down-regulate the function of *Serpinc1* gene by 70% using LNP-based delivery of the CRISPR-Cas9 editing system and improved thrombin generation in both hemophilia A and B mouse models without reported off target effects. Their results showed that unlike viral vectors, repeated *in vivo* application of LNPs is not problematic in terms of induction of immune response. Intellia Therapeutics in cooperation with Regeneron have developed a CRISPR-mediated treatment research program for hemophilia A and B. In this program, they use LNPs to insert a transgene in the liver of non-human primates to produce human Factor IX, which is necessary to treat hemophilia A and B (https://www.intelliatx.com/our-pipeline/). In another study, Ho et al. (2021) delivered bioreducible lipidoid-encapsulated Cas9-sgRNA into human leukemia stem cells (LSCs) to knock-out interleukin-1 receptor accessory protein (IL1RAP). It led to decreased clonogenicity of leukemia cells *in vitro* and reduced leukemic burden *in vivo*.

Inorganic nanoparticles, in particular gold nanoparticles (AuNPs), are another interesting option for delivery of genetic materials into the cells. These kinds of nanoparticles can be adaptable to different sizes and chemical modifications and can be applicable in combining with lipids or polymers. Moreover, they are less toxic for cells than lipid and polymer nanocarriers (Ding et al., 2014; Behr et al., 2021). Shahbazi et al. (2019) developed a gold nanoparticles-based delivery for CRISPR gene editing system in HSPCs. The multilayered AuNP/CRISPR nanoformulation used by this group could be detected by confocal microscopy imaging 6h after treatment in HSPCs. They could also target γ-globin promoter on chromosome 11 and reach 12.1% total editing in this region. The result showed no impact on colony formation and xenograft analysis after infusion of *ex vivo* edited CD34^+^ HSPC into neonatal immune-deficient mice.

Polymer-based nanoparticles use the same strategy as LNPs for delivery of CRISPR components in different forms through the cell membrane. Polymeric NPs have high stability and capacity for cargo encapsulation (Zielińska et al., 2020; Piperno et al., 2021). El-Kharrag et al. (2022) compared the polymer-based nanoparticles delivery and electroporation of mRNA and nucleases into human granulocyte colony-stimulating factor (GCSF)-mobilized CD34^+^ cells with electroporation method. They found that despite similar efficiency, polymer NPs based delivery needs three times less reagents than electroporation. They also proposed PBAE-NPs as an efficient method for CRISPR-Cas9 gene editing in HSPCs *in vivo*.

### Viral delivery

Different viral vectors have been used for the delivery of CRISPR components as natural delivery systems. Adeno-associated virus (AAV) is a parvovirus with no report of causing disease in human (Lau and Suh, 2017). Mitochondrial DNA and AAVS1 site on chromosome 19 are known as integration sites for AAV, although they are considered safe locations without the risk of tumorigenesis (Kaeppel et al., 2013; Xu et al., 2019). Besides natural AAVs, recombinant AAV vectors are also designed with the aim of increasing the transduction efficiency and decreasing immune response (Li and Samulski, 2020). Because of their safety and therapeutic potential, AAVs are attractive vehicles for *in vivo* delivery of gene editing elements into a wide range of cells. AAV-CRISPR mediated gene therapy is a promising approach for the treatment of blood disorders especially hemophilia. Different studies have assessed the efficiency of AAVs as a delivery method for gene editing in hemophilia A and B *in vitro* and *in vivo* (Chen et al., 2019; Gao et al., 2019; Zhang et al., 2019; Wang et al., 2020).

CRISPR components can be transferred to the cells using either single or dual viral vectors. Gao et al. (2019) compared these two methods for gene editing in hemophilia B cells (Huh7-cFIXmut cells). In one strategy, they transduced cells with adenovirus vector type 5 (HCAdV5) carrying CRISPR-Cas9 and single-stranded adeno-associated virus type 2 vector (ssAAV2) carrying modified donor. In the second strategy, they utilized a single HCAdV5 for the delivery of all components of repair. They found that single vector application is more efficient than two vectors. Although Wang et al. (2020) showed that dual AAV vectors (AAV8.SpCas9 and AAV8.sgRNA. donor vector) application *in vivo*, is a safe method to integrate partial human *FIX* (*hFIX*) in mouse *albumin* (*mAlb*) and enhance the coagulation in hemophilia B mice.

Chen et al. (2019) used two recombinant AAV8 vectors containing Cas9-gRNA and codon-optimized human B-domain-deleted human FVIII (*BDD-F8*) encoding sequence to insert *BDD-F8* at liver-specific albumin (*Alb*) locus in hemophilia A mouse model. This treatment resulted in the improvement of the disease phenotype and increasing of FVIII protein and activity levels in mice liver without toxicity for 7 months. Different studies have employed AAVs as delivery method for the treatment of human immunodeficiencies such as chronic granulomatous disease (Sweeney et al., 2017) and human immunodeficiency virus (HIV) infection (Sather et al., 2015; Yin et al., 2017; Dash et al., 2019). Nahmad et al. (2022) succeeded in *in vivo* engineering of B cells using AAV vectors (dual AAV-DJ) for the delivery of CRISPR and the production of therapeutic antibodies against HIV in mice. Recently, Excision Bio Therapeutics has started a phase 1/2 clinical trial evaluating EBT-101, a CRISPR-Cas9 based therapy, using AAV9 for delivery through intravenous (IV) administration in aviremic HIV-1 infected adults (NCT05144386). AAV vectors are not the optimal delivery methods for all cell types because the viral genome can remain in the cells as an episome and AAV capsid proteins may lead to immune responses (Park et al., 2016; Chen et al., 2019).

Adenoviral vectors (AVs) have higher capacity (>8 kB) which makes possible a single transferring of Cas9 and sgRNAs in only one vector. AVs can be delivered into both dividing and non-dividing cells without integration in the host genome, nevertheless, there is a high risk of induction of immune response in cells after AVs transduction (Wilbie et al., 2019; Yip, 2020). Guan et al. (2016) compared two different strategies including naked DNA constructs and adenoviral vectors for the delivery of Cas9 component to hemophilia B mice models carrying F9 Y371D mutation. They reported that although using adenoviral delivery system leads to higher editing efficiency than naked DNA, it also shows severe hepatic toxicity. Helper-dependent Ad (*HDAd*) is the recombinant form of AVs in which all viral genome is deleted except the packaging sequence and cis-acting ITRs, contributing to high transgene capacity (Vetrini and Ng, 2010; Rosewell Shaw et al., 2022). *HDAd* expressing CRISPR-Cas9 is utilized to reactivate human γ-globin by disrupting the repressor binding site in γ-globin promoter in HSPCs and β-YAC/CD46–transgenic mice (Li et al., 2018). The result showed an increase of the HBG/HBB expression ratio *ex vivo* and *in vivo* without negative effect on hematopoiesis (Li et al., 2018). A subsequent study has utilized HSPCs transduction with *HDAd* vectors expressing CRISPR and globin donor *ex vivo* and *in vivo* and could reach stable levels of γ-globin expression (Li et al., 2019).

Retroviral vectors including gamma-retrovirus and lentivirus vectors are also used by scientists as gene therapy tools (Ghosh et al., 2020). Lentiviral vector systems are interesting especially for *ex vivo* gene editing because of their high capacity for carrying complex transgenes and their ability to express in both dividing and non-dividing cells (Lattanzi et al., 2019; Gutierrez-Guerrero et al., 2020; Dong and Kantor, 2021). Therefore, these vectors are considered efficient methods for the delivery of CRISPR components into the cells. However, long-term expression and high frequency of off target effects due to the integration of LVs into the genome is the biggest limitation of these vectors. Lentiviral based CRISPR delivery system is used for the disruption of different genes involved in blood disorders. Silencing of mucin 1 C-terminal subunit (*MUC1-C*), an oncogenic transmembrane protein, by CRISPR-Cas9 promotes the reduction of *MYC* oncoprotein expression and β-catenin levels in multiple myeloma (MM) cells (Daniel and Molta, 1989). Gene disruption in the HBG1/HBG2 promoter sequence by lentivirus expressing Cas9 and guide RNA increased HbF levels in CD34^+^ cells to simulate a natural benign condition that prevents the symptoms of HBB mutation in SCD and β-thalassemia (Traxler et al., 2016).

Using lentivirus for introducing edited *ABL* gene into leukemia xenograft mouse model showed significant inhibition of leukemia cell growth (Chen et al., 2020). Further on, Chen et al. (2020) transduced patient CML cells *ex vivo* with a lentivirus based CRISPR-Cas9 genome editing system and obtained more than 30.9% indel frequency without reporting off-target effects. They could show that the *ABL*-targeted CRISPR-Cas9 virus can lead to a high rate of apoptosis in CML cells. On the contrary, different studies have shown that gammaretroviral vectors-mediated gene therapy approaches in hematopoietic stem cells can result in serious side effects such as leukemogenic risks (Hacein-Bey-Abina et al., 2003a; Hacein-Bey-Abina et al., 2003b; Ott et al., 2006; Stein et al., 2010; Braun et al., 2014).

Due to the increasing concern about the inevitable risk of insertional mutagenesis, vector design has improved, and new generations of SIN gamma- and lentiviral vectors with inactivated LTRs have been developed to minimize the oncogenic potential and improve their use in clinical settings (Engelman et al., 1995; Hacein-Bey-Abina et al., 2014; Shaw and Cornetta, 2014; Daniel-Moreno et al., 2020). These modified viruses will harbor non-replicating episomes, which have certain limitations such as loss over time in rapid-dividing cells, lower transgene expression in comparison to traditional lentiviruses and risk of residual integration (Luis, 2020; Gurumoorthy et al., 2022). Nevertheless, integrase defective lentiviruses (IDLVs) can effectively transduce HSPCs and have proven to be effective donor template carriers in preclinical studies of X-linked severe combined immunodeficiency (X-SCID) therapy (Genovese et al., 2014) and CRISPR-Cas9 deliverer for the repair of patient-specific frameshift point mutations (CYBB) involved in chronic granulomatous disease (XCGD) (Sürün et al., 2018).

## Discussion

In recent years, the emergence and development of the CRISPR-Cas system have made a great revolution in genome editing technology. Pre-clinical and clinical results of applying this technique for the treatment of different genetic disorders are very promising. On target and off target cutting, homology directed repair efficiency, proper guide RNA and donor template selection and suitable delivery method are critical considerations in CRISPR based gene editing (Lino et al., 2018). In this review, we summarized the state of art for the delivery methods of the CRISPR components into the cells with a focus on gene editing in blood cells. It has been shown that despite many attractive features and high gene editing efficiency, viral vectors have significant limitations. Recent findings concerning the integration of AVV vectors into the CRISPR induced DSBs sites have questioned the safety of these vectors for CRISPR-based gene therapy (Hanlon et al., 2019). The induction of immunogenicity and cellular toxicity by some types of adenovirus and the risk of insertional mutagenesis by lentiviral vectors is still challenging for using these vectors in gene editing (Bulcha et al., 2021). Physical methods are likely to be the most convenient method for *ex vivo* therapy development but are not feasible for treatments based on gene editing *in vivo*, where non-carriers such as lipid or polymer-based NPs seems to have a brighter future. Overall, we believe that the substantial progress and optimization in current delivery options will promote the CRISPR-Cas9 application for the treatment of blood disorders in the coming decades.
